# Calcium-binding proteins as allergens

**DOI:** 10.3389/falgy.2026.1759312

**Published:** 2026-02-02

**Authors:** Andrea O'Malley, Kriti Khatri, Elaine M. Wright, Rebekka A. Pittsley, Krzysztof Kowal, Maksymilian Chruszcz

**Affiliations:** 1 Department of Biochemistry and Molecular Biology, Michigan State University, East Lansing, MI, United States; 2 Department of Experimental Allergology and Immunology, Medical University of Bialystok, Bialystok, Poland; 3 Department of Allergology and Internal Medicine, Medical University of Bialystok, Bialystok, Poland

**Keywords:** allergen, calcium, cross-reactivity, EF-hand, IgE, stability

## Abstract

Calcium-binding proteins, particularly those in the EF-hand family, are found ubiquitously in nature, primarily for calcium transport and storage in the body. In this review, we discuss allergens in the parvalbumin, polcalcin, sarcoplasmic calcium-binding protein, and troponin C families, as well as additional allergens. Allergens from these protein families display a wide range of IgE reactivity and cross-reactivity. They are implicated in both inhaled and food allergies, and, due to their common presence, they are difficult to avoid.

## 1 Introduction

Calcium is essential for all areas of life, including muscle contraction, blood cell synthesis and function, and nerve conduction. In eukaryotes, the primary calcium-binding protein family is the EF-hand family, which is characterized by the EF-hand, a helix-loop-helix motif composed of 29–30 amino acids (1). The EF-hand motif is featured heavily in cellular signaling as it facilitates calcium movement (2). However, the specific function of each protein family will vary based on the structure, expression pattern, and cellular location of the protein. The EF-hand superfamily is particularly diverse since some proteins are composed solely of the EF motif while other proteins have the motif joined to the rest of the protein (1).

Many protein families in the EF-hand superfamily contain allergens (Table 1). Included in these families are parvalbumins (PV), polcalcins, sarcoplasmic calcium-binding proteins (SCPs), and troponin C (TnC). All of these protein families contain important allergens that cause significant reactions in individuals allergic to various organisms, including seafood, pollen, and mites (3). Two additional protein families, psoriasin (S100A7) and mitochondrial calcium-binding protein, each contain one registered allergen that have not been studied in depth.

**Table 1:** Calcium-binding allergens registered in the wHO/IUIS (23).

Protein family	Allergen	Organism (Common Name)	Route
Parvalbumin	Clu h 1	*Clupea harengus* (Atlantic herring)	Ingestion
	Cro p 1	*Crocodylus porosus* (saltwater crocodile)	Ingestion
	Cro p 2	*Crocodylus porosus* (saltwater crocodile)	Ingestion
	Cten i 1	*Ctenopharyngodon idella* (grass carp)	Ingestion
	Cyp c 1	*Cyprinus carpio* (common carp)	Ingestion
	Gad c 1	*Gadus callarias* (Baltic cod)	Ingestion
	Gad m 1	*Gadus morhua* (Atlantic cod)	Ingestion
	Gal d 8	*Gallus domesticus* (chicken)	Ingestion
	Late m 1	*Lateolabrax maculatus* (Asian spotted seabass)	Ingestion
	Lat c 1	*Lates calcarifer* (barramundi)	Ingestion
	Lep w 1	*Lepidorhombus whiffiagonis* (whiff)	Ingestion
	Onc m 1	*Oncorhynchus mykiss* (rainbow trout)	Ingestion
	Pan h 1	*Pangasianodon hypophthalmus* (striped catfish)	Ingestion
	Ran e 1	*Rana esculenta* (edible frog)	Ingestion
	Ran e 2	*Rana esculenta* (edible frog)	Ingestion
	Ras k 1	*Rastrelliger kanagurta* (Indian mackerel)	Ingestion
	Sal s 1	*Salmo salar* (Atlantic salmon)	Ingestion
	Sar sa 1	*Sardinops sagax* (Pacific pilchard)	Ingestion
	Sco s 1	*Scomber scombrus* (Atlantic mackerel)	Ingestion
	Seb m 1	*Sebastes marinus* (ocean perch)	Ingestion
	Sole s 1	*Solea solea* (sole)	Ingestion
	Thu a 1	*Thunnus albacares* (yellowfin tuna)	Ingestion
	Tric l 1	*Trichiurus lepturus* (Atlantic cutlass)	Ingestion
	Xip g 1	*Xiphias gladius* (swordfish)	Ingestion
Polcalcin	Aln g 4	*Alnus glutinosa* (alder)	Inhalation
	Amb a 9	*Ambrosia artemisiifolia* (short ragweed)	Inhalation
	Amb a 10	*Ambrosia artemisiifolia* (short ragweed)	Inhalation
	Art d 5	*Artemisia desertorum* (desert wormwood)	Inhalation
	Art si 5	*Artemisia sieversiana* (Sierversiana wormwood)	Inhalation
	Art v 5	*Artemisia vulgaris* (mugwort)	Inhalation
	Bet v 3	*Betula pendula* (European white birch)	Inhalation
	Bet v 4	*Betula pendula* (European white birch)	Inhalation
	Bra r 5	*Brassica rapa* (turnip)	Inhalation
	Che a 3	*Chenopodium album* (lamb's quarters)	Inhalation
	Cyn d 7	*Cynodon dactylon* (Bermuda grass)	Inhalation
	Jun o 4	*Juniperus oxycedrus* (prickly juniper)	Inhalation
	Sal k 7	*Salsola kali* (prickly saltwort)	Inhalation
	Ole e 3	*Olea europaea* (olive)	Inhalation
	Ole e 4	*Olea europaea* (olive)	Inhalation
	Par j 4	*Parietaria judaica* (pellitory of the wall)	Inhalation
	Phl p 7	*Phleum pratense* (timothy grass)	Inhalation
	Syr v 3	*Syringa vulgaris* (lilac)	Inhalation
Sarcoplasmic calcium binding protein	Aed a 5	*Aedes aegypti* (yellow fever mosquito)	Inhalation
	Cra c 4	*Crangon crangon* (North Sea shrimp)	Ingestion
	Cra a 4	*Crassostrea angulata* (Portuguese oyster)	Ingestion
	Lit v 4	*Litopenaeus vannamei* (white shrimp)	Ingestion
	Pen m 4	*Penaeus monodon* (black tiger shrimp)	Ingestion
	Pon l 4	*Pontastacus leptodactylus* (narrow-clawed crayfish)	Ingestion
	Por t 4	*Portunus trituberculatus* (Gazami crab)	Ingestion
	Scy p 4	*Scylla paramamosain* (green mud crab)	Ingestion
Troponin C	Bla g 6	*Blattella germanica* (German cockroach)	Inhalation
	Cra c 6	*Crangon crangon* (North Sea shrimp)	Ingestion
	Der f 39	*Dermatophagoides farinae* (American house dust mite)	Inhalation
	Der p 39	*Dermatophagoides pteronyssinus* (European house dust mite)	Inhalation
	Hom a 6	*Homarus americanus* (American lobster)	Ingestion
	Pen m 6	*Penaeus monodon* (black tiger shrimp)	Ingestion
	Per a 6	*Periplaneta americana* (American cockroach)	Inhalation
	Pon l 7	*Pontastacus leptodactylus* (narrow-clawed crayfish)	Ingestion
	Tyr p 34	*Tyrophagus putrescentiae* (storage mite)	Inhalation
S100 calcium-binding protein A7	Bos d 3	*Bos taurus* (domestic cattle)	Inhalation
Mitochondrial calcium uptake protein	Hom s 4	*Homo sapiens* (human)	Autoallergen

In this review, we discuss the function, structure, and immunological features of proteins from these families. Notably, we focus on the immunological impact of calcium-binding on these selected allergens and the allergens’ IgE cross-reactivity. Their stability and cross-reactivity make them highly potent allergens that should be carefully studied and avoided by allergic individuals.

## 2 Calcium-binding allergens

### 2.1 Parvalbumin

**Figure 1:**
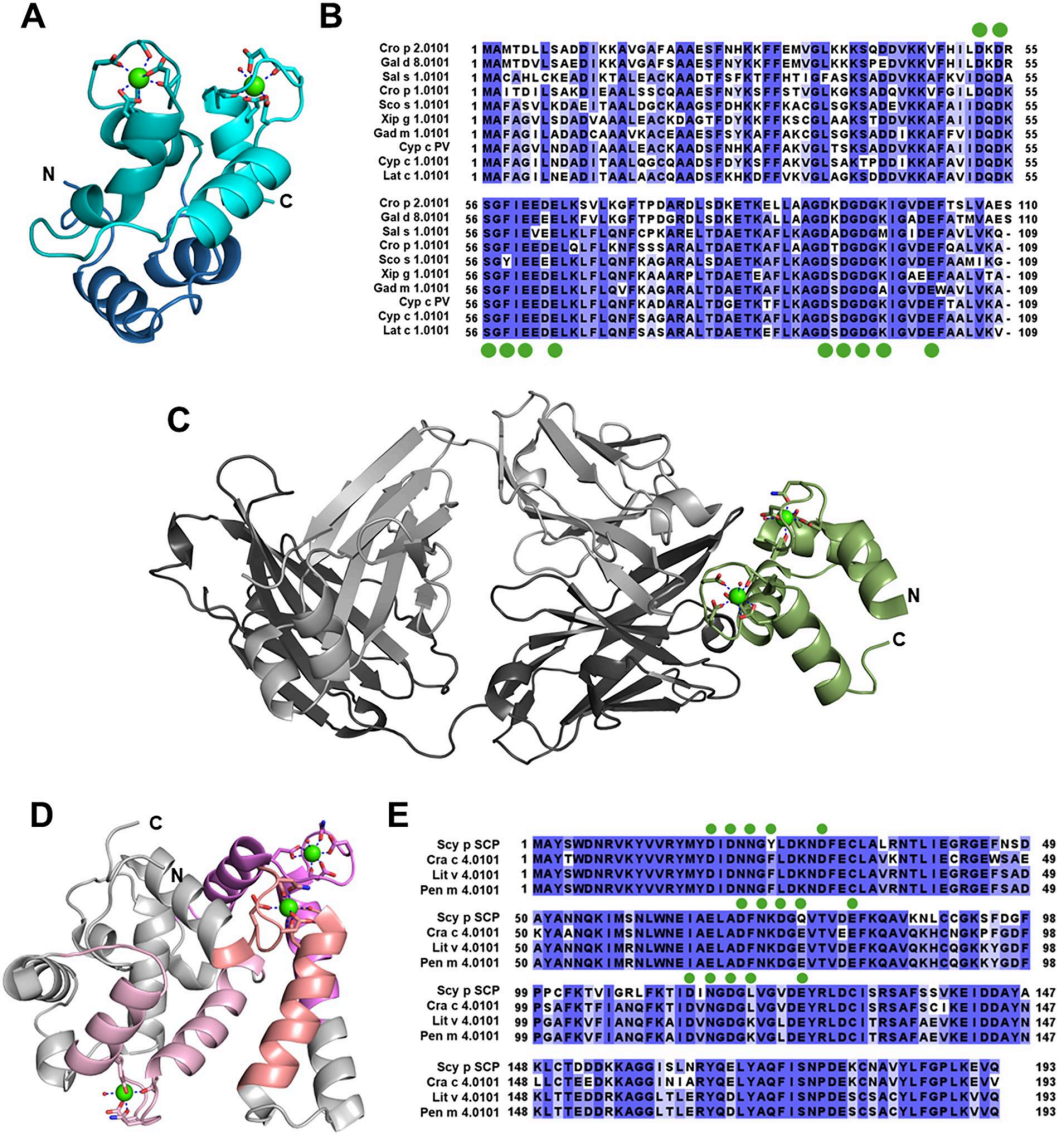
**(A)** carp β-parvalbumin (PDB: 5CPV) with residues involved in binding calcium (green spheres) shown as sticks. The EF-1 (AB) motif is shown in dark blue, the EF-2 (CD) motif in teal, and the EF-3 (EF) motif in cyan; domain division is as predicted on Uniprot. N- and C-terminals are labeled, and the water molecule that completes the EF motif is shown as a red sphere. Bonds between calcium and coordinating residues are shown as navy dashes. **(B)** Sequence alignment of selected PV allergens as well as the non-registered carp β-PV (PDB: 5CPV). Residues that coordinate calcium are indicated with green circles. **(C)** Structure of Phl p 7 (olive green) in complex with 102.1F10 Fab. The light chain of the Fab is colored light gray, with the heavy chain in dark gray. The residues coordinating calcium (green spheres) in Phl p 7 are shown as sticks, the bonds are shown as navy dashes, and the water molecules are shown as red spheres. N- and C-terminals are labeled accordingly. **(D)** An isoform of Scy p 4 (PDB: 7WBO) with residues involved in binding calcium (green spheres) shown as sticks. The EF-1 motif is shown in Salmon, EF-2 in violet, and EF-3 in pink; domain division is as predicted on Uniprot. N- and C-terminals are labeled, and the water molecules that complete the motifs are shown as red spheres. Bonds between calcium and coordinating residues are shown as navy dashes. **(E)** Sequence alignment of selected SCP allergens, as well as the Scy p 4 isoform (PDB: 7WBO). Residues that coordinate calcium are indicated with green circles.

Parvalbumin (PV) is considered a prototypic EF-hand calcium-binding protein (4). PV is primarily found in the muscle tissue of mammals and marine organisms as well as in neural tissue, although its exact function is unclear (5). PVs (11–13 kDa) have three EF-hand motifs, two of which bind calcium cations (Figure 1A); the AB hand has a two-residue deletion that prevents calcium binding (6). Recombinant PV is stable in high temperatures, and natural PV tends to survive cooking processes. PV is prone to unfolding and aggregating at acidic pH levels, which is consistent with reduced calcium coordination under acidic conditions (7–9).

PVs are categorized as either alpha-PV (α-PV) or beta-PV (β-PV) based primarily on the protein sequence and their sizes (10). β-PV is well-known as a major vertebrate seafood allergen, with 70%–100% of vertebrate seafood-allergic individuals having reactions, and has been studied in Atlantic cod (Gad m 1), carp (Cyp c 1), salmon (Sal s 1), and saltwater crocodile (Cro p 1), among other organisms (11–15). Bony fish have both α-PV and β-PV, while cartilaginous fish have only α-PV. Interestingly, β-PVs are highly cross-reactive with each other (average sequence identity of 70% across registered allergens) but not usually with α-PVs; this is likely caused by the lower sequence identity (approximately 50%) between the two isoforms (16). α-PVs are also rarely classified as allergens due to low IgE reactivity (14). The calcium binding residues on the EF-hands are highly conserved (Figure 1B), making the lack of cross-reactivity and varied IgE binding between the two isoforms more interesting (10). Individuals with seafood allergies to bony fish will likely have significant reactions to β-PV, including allergic responses caused by protein cross-reactivity.

PV has been extensively structurally characterized with both x-ray crystallography and NMR. Molecular evaluation of PV has shown extreme structural conservation among the protein family. All x-ray structures of wild-type PV have two metal cations bound, demonstrating the proteins’ reliance on metal cations for stability. Some x-ray structures have been determined for mutant PV, with residues in the CD or EF motif mutated to prevent calcium binding (4), and some NMR structures have been solved for apo wild-type PV (17, 18). Calcium-depleted PV (Cyp c 1 mutant) has been proposed as a hypoallergen for treatment of carp allergy, with an average of 1,000-fold allergenic activity lost in the mutant (19, 20).

### 2.2 Polcalcin

Polcalcins are found in grass, weed, and tree pollen and are considered minor allergens. The primary functions of polcalcin are signaling between plant cells and pollen tube growth via calcium transport (21, 22). There are two categories for polcalcins, two EF-hand proteins and four EF-hand proteins, with one of each having been identified for some plants. For example, ragweed has both Amb a 9 (two EF-hands) and Amb a 10 (four EF-hands), and silver birch has Bet v 3 (two EF-hands) and Bet v 4 (four EF-hands) (23). The two-EF hand polcalcins are approximately 9 kDa, while the four EF-hand polcalcins are up to 17 kDa. Polcalcins have highly conserved sequences, particularly in the calcium binding residues and are particularly cross-reactive (3, 24).

Structural characterization of polcalcin has shown that calcium coordination will significantly affect the folding of the protein. Structural variation was revealed by NMR structures of Phl p 7 (timothy grass) in the apo form or when bound to one or two calcium cations (22). Interestingly, two structures of domain-swapped polcalcin were solved, one of Phl p 7 and one of Che a 3 (lamb's quarters). These structures may be the result of the acidic crystallization condition (pH 3.3–3.4), rather than representing the physiological polcalcin, thus demonstrating the effect of calcium coordination and movement on the protein structure (25, 26).

Based on the structures of polcalcin with and without calcium, it can be assumed that *apo* polcalcin will have a significantly different shape in solution as compared to *holo* polcalcin, leading to varied IgE interaction from each protein. This was shown with Ole e 3 (olive), where the addition of calcium did not affect IgE binding, but the addition of a chelating agent lowered IgE binding by 32% (27). It was also demonstrated that calcium removal decreased the affinity of a specific human IgE to Phl p 7 over 10,000-fold (28). The structure of Phl p 7 bound to the antibody was the first structure of a specific antibody bound to a calcium-binding allergen (Figure 1C). Additionally, a version of Phl p 7 was generated as a potential hypoallergen, with two residues in each calcium binding site mutated to alanine to prevent calcium binding (29). The authors also demonstrated that calcium-depleted wild-type Phl p 7 and mutant Phl p 7 had similar structures and *α*-helical content to the wild-type Phl p 7, suggesting that the secondary structure of polcalcin may not be reliant on calcium binding.

### 2.3 Sarcoplasmic calcium-binding protein

Sarcoplasmic calcium-binding proteins (SCPs) are widely present in crustaceans, mollusks, and insects. With three EF-hand motifs that bind three calcium cations per protein molecule, they play a role in muscle calcium regulation (30–32). Their molecular architecture imparts thermal stability, maintaining allergenicity even after food processing (30, 32). Some SCPs, such as Lit v 4 (white shrimp), retain the ability to trigger mast cell and basophil activation at temperatures up to 80°C, and its structural retention after heating was shown with Circular Dichroism spectroscopy (30, 33).

Lit v 4, Por t 4 (Gazami crab), and Scy p 4 (mud crab) are major allergens, eliciting IgE responses in 30%–60% of shellfish-allergic individuals, particularly among children (33–36). Pon l 4 (narrow-clawed crayfish) and Cra a 4 (Portuguese oyster) are also considered important food allergens (31, 37, 38). Minor SCP allergens such as Cra c 4 (North Sea shrimp) and Pen m 4 (black tiger shrimp) have sensitization rates of 10%–15% in European shrimp-allergic patients, but these rates can reach over 50% in some populations (37, 39, 40). Investigation of SCP sensitization in unique populations has suggested that SCPs contribute to broader patterns of shellfish allergy and occasionally cause severe symptoms independently of sensitization to tropomyosin or another major allergen (39). Interestingly, SCPs can also act as inhalant allergens via occupational exposure. Shellfish SCPs can provoke symptoms such as asthma and dermatitis in processing workers, while respiratory and cutaneous reactions result from airborne or contact exposure, not ingestion alone (41).

The structure of Scy p 4 was determined, allowing for evaluation of IgE binding epitopes and confirming the calcium-binding sites (Figure 1D). Site-directed mutagenesis or deletions at key calcium-binding residues resulted in a significant reduction or loss of allergenicity (42, 43). SCPs from different species share high structural homology, which lends itself to IgE cross-reactivity (43). Cross-species sequence analysis (Figure 1E) and epitope mapping have demonstrated significant overlap, especially between shrimp, crab, and crayfish SCPs, explaining the frequency of allergic reactions spanning multiple shellfish groups (31, 44). SCP-specific IgE rarely cross-reacts with non-crustacean SCPs, but strong reactivity is seen among crustacean SCPs due to conserved epitopes (31, 44, 45).

### 2.4 Troponin C

Troponin C (TnC) is a calcium-binding subunit of the troponin complex, a multiunit protein complex essential for skeletal and cardiac muscle contraction (46–49). Structurally, TnC is a dumbbell-shaped globular protein formed largely by *α*-helices. The two globular domains, each with two EF-hand motifs, are at the N- and C-terminals and are connected by a long flexible linker (50). TnC in vertebrates and invertebrates exhibit some diversity in their calcium-binding EF-hand motifs and overall primary structure, although their structure-function relationship is conserved (51). In vertebrate TnC, the four EF-hands each have varying affinity for calcium or magnesium cations (52). In invertebrates, the functional calcium binding sites are more diversified (53). For example, crustacean and nematode TnCs bind two calcium cations, molluscan TnCs binds one calcium cation, and TnCs from cockroach and storage mite are each predicted to bind two calcium cations (53–55).

TnC has been identified as a minor allergen in several invertebrates, including mites, cockroaches, and crustaceans such, as shellfish (45). IgE against Pen m 6 (Indian black tiger shrimp) has been observed among 47.1% and 50.0% of shrimp-allergic populations from Hong Kong and Thailand respectively (56). Similarly, studies have shown binding among 29% of sera to Cra c 6 (North Sea shrimp) and IgE binding from 24% of patients to Hom a 6 (American lobster) (57, 58). Bla g 6 (cockroach), Tyr p 24 (storage mite), and Der p 39 (house dust mite) are minor inhalant allergens with positive IgE testing in 16.7%, 10.6%, and 5.3% of studied populations, respectively (54, 55, 59).

Currently, there are no experimental structures of TnC allergens available in the Protein Data Bank for any invertebrate TnC proteins (60). TnC can undergo conformational changes upon calcium binding, which may conceal or reveal IgE binding epitopes; this was observed with Tyr p 24, where the addition of calcium increased IgE binding (55). A conformational epitope for Der p 39 has been demonstrated in the C-terminal of the protein (59). TnC is also a heat-stable allergen, with IgG binding capacity retained at 100°C for the TnC from *Scylla paramamosai* (56).

Allergic cross-reactivity has been predicted for Per a 6 and Bla g 6 (54). Tyr p 24 shares 63%–86% sequence homology with TnCs from different arthropods, which may lead to IgE cross-reactivity (55). Sequence diversity, mostly in calcium binding sites, have been found across TnCs in some species and even across isoforms of same species. The three isoforms of Bla g 6, which share 68%–91% sequence identity, exhibited a similar IgE response, suggesting that major IgE epitopes are mostly in the identical regions of the protein (54).

### 2.5 Psoriasin (S100 calcium-binding protein A7)

Psoriasin is an S100 calcium-binding protein (S100A7). It is expressed in the cytoplasm of epithelial cells and contains two EF-hand motifs, binding one calcium molecule per protein molecule (61). It is expressed as a homodimer and has a molecular weight of 11.4 kDa (62). Psoriasin has intracellular and extracellular functions, including cell proliferation, differentiation, apoptosis, calcium transduction, and energy metabolism (63). Psoriasin expression is influenced by inflammatory stimuli and oxidative stress via reactive oxygen species and hypoxia, and increased levels of psoriasin have been associated with chronic inflammatory conditions, such as atopic dermatitis and psoriasis (63, 64).

Bos d 3 is a psoriasin allergen from cow dander with 64% sequence identity with human psoriasin. Little research has been conducted into the allergenicity of Bos d 3 independently of its homology to human psoriasin (61, 65). However, it may be an antimicrobial agent in cow's milk (65, 66). It is suggested that, like human psoriasin, Bos d 3 binds zinc with conserved histidine residues that bridge the dimer (61). Further investigation is needed to evaluate the potential cross-reactivity of Bos d 3 with human psoriasin, as well as with other mammalian homologues.

### 2.6 Mitochondrial calcium-binding protein

Hom s 4 is an autoantigen allergen involved in atopic dermatitis (67). It is classified as a mitochondrial calcium-binding protein, with a molecular weight of 54 kDa, and it contains two EF-hand motifs separated by an *α*-helical domain (67, 68). Hom s 4 is ubiquitously present, located in the mitochondria of epithelial cells, especially keratinocytes (67). It induces Th1-mediated autoreactivity via IFN-γ release (68). Some research suggests that this autoreactivity arises from the epitope sequence of Hom s 4 binding to HLA-DRB1 (67). It has been shown that individuals with atopic dermatitis that had IgE reactivity to Hom s 4 (43% of a tested population) were more likely to have reactions to other allergens, including drugs, pollen, mites, fungi, foods, and animals (69).

## 3 Discussion

In this work, we have discussed allergens from six protein families that contain calcium binding domains, all of which are the EF-hand motif. PVs, polcalcins, SCPs, and TnCs, the allergens have a wide range of IgE reactivity, allergic cross-reactivity, and structural response to calcium coordination. Significantly less research has been performed into allergens from the psoriasin and mitochondrial calcium-binding protein families, but they are interesting cases in the effect of human calcium binding proteins on allergy.

Additional allergens have been suggested or shown to bind calcium, but the relevance of the calcium binding in IgE response to the allergen has not been confirmed. The allergens also do not contain the EF-hand motif expected of calcium binding allergens. Major house dust mite allergens Der f 1 and Der p 1 are homologous cysteine proteases (70). Structurally, Der f 1 and Der p 1 exhibit a papain-like fold with two domains that form the catalytic site for the protease activity (71). The structure of Der p 1 (PDB: 3F5V) also reveals a calcium cation bound distant from the catalytic site (71). While the function of calcium in Der f 1 or Der p 1 is not clear, it may play a role in ligand binding and have an indirect effect on catalytic activity (70). Three putative calcium binding sites have also been reported for major cat allergen Fel d 1 as shown in the published structure of Fel d 1 (PDB: 2EJN) (72). However, molecular dynamics studies have suggested that there is only one physiological calcium binding site in Fel d 1, which may have a role in stability of the tetrameric assembly of the protein (73).

A commonality between the allergens discussed in this work is their stability. PVs, SCPs, and TnCs retain their structure and IgE-binding capacity at high temperatures, leading to the possibility or likelihood that the proteins will survive food processing. However, most calcium-binding proteins do not retain their folds in conditions with low pHs (more acidic than pH 3). It is possible that they will refold when exposed to more basic conditions. Such refolding was shown to be possible in the case of non-specific lipid transfer protein Cor a 8 (74). Another possibility is that the aggregates formed upon protein unfolding will have different IgE-binding epitopes that still cause an allergic reaction. Thus, reactions to any of these proteins cannot be discounted, even when the food is cooked.

Additionally, most of the proteins discussed in this work display significant IgE-mediated cross-reactivity. For example, it was previously demonstrated that seafood-allergic individuals may have reactions to reptile PVs (10). Similarly, individuals with house dust mite allergy caused by sensitization to TnC may have oral allergy syndrome-like symptoms to seafood (55). The allergens from each family exhibit up to 80% sequence identity with other members of the protein family, which strongly suggests that cross-reactivity is likely. Additionally, the residues generating the calcium-binding sites within the EF-hands are often similar, leading to specific cross-reactivity on the EF-hand motifs. Thus, calcium-binding allergens in the EF-hand family are particularly prone to causing IgE-mediated cross-reactivity, making them particularly troublesome allergens.

In summary, calcium-binding allergens from the EF-hand family, including parvalbumins, polcalcins, sarcoplasmic calcium-binding proteins, and troponin C pose a significant threat to individuals with seafood, pollen, and mite allergies. They are generally thermally stable and may generate IgE-mediated cross-reactivity. Additionally, psoriasins and mitochondrial calcium-binding proteins may cause allergic reactions, possibly caused by cross-reactivity with human counterparts. Overall, individuals with reactions to one protein from these families should carefully consider other potential allergens, since additional reactions are likely to occur upon contact.
